# The use of propensity score matching to assess the effectiveness of the endometrial receptivity analysis in patients with recurrent implantation failure

**DOI:** 10.3389/fendo.2024.1402575

**Published:** 2025-01-13

**Authors:** Shuang Yu, Yongjie Zhang, Na Li, Zhuolun Su, Wenjing Li, Hua Lou, Yichun Guan

**Affiliations:** ^1^ Reproductive Center, The Third Affiliated Hospital of Zhengzhou University, Zhengzhou, China; ^2^ Reproductive Center, The Second Hospital of Lanzhou University, Lanzhou, China

**Keywords:** endometrial receptivity analysis, frozen embryo transfer, *in vitro* fertilization, propensity score matching, recurrent implantation failure, personalized embryo transfer

## Abstract

**Background:**

One potential cause of implantation failure is abnormal endometrial receptivity, and how to objectively evaluate endometrial receptivity has been a matter of great concern. Endometrial receptivity analysis (ERA), a next-generation sequencing-based test that assesses endometrial gene expression, may be valuable in predicting endometrial receptivity, but whether ERA improves pregnancy outcomes in patients with recurrent implantation failure (RIF) is currently controversial. The purpose of this study was to investigate the effect of ERA on pregnancy outcomes in patients with RIF.

**Methods:**

We performed a retrospective cohort study analysis for a population of patients with RIF undergoing frozen embryo transfer (FET) cycles in the reproductive center of the Third Affiliated Hospital of Zhengzhou University from January 2019 to December 2022(n=1598). FET cycles with personalized embryo transfer (PET) under ERA guidance were included in the ERA group (n=43); after using propensity score matching (PSM), a total of 120 FET cycles were included as a control group. Pregnancy outcomes were compared between the two groups. Further, the relationship between the number of previous implant failures and the rate of implant window displacement was discussed. The factors affecting the window of implantation (WOI) displacement were also assessed.

**Results:**

There was no statistically significant difference in embryo implantation rate, clinical pregnancy rate, spontaneous abortion rate, and live birth rate between the ERA group and the matched control patients (*P* > 0.05). There was no significant difference in the rate of WOI displacement between patients in the moderate or severe groups (*P* > 0.05) and no significant difference in pregnancy outcome ( *P*>0.05). Finally, analysis of the clinical data of patients in the receptive and non-receptive groups did not uncover any factors influencing WOI displacement.

**Conclusion:**

The results of the study showed no significant difference in pregnancy outcomes in patients who received ERA compared to those who did not.

## Introduction

Reproductive medicine has made many advances and achievements over the past 40 years, but recurrent implantation failure (RIF) remains a challenging problem (1, 2). There is a lack of consensus on the definition of RIF, with the approximate prevalence of RIF ranging from 10%~20% (1, 3, 4). RIF can be a huge financial and psychological burden for patients, so there is an urgent need to search for the cause of RIF and for treatments that can improve their pregnancy outcomes. RIF involves complex etiologies (2, 5, 6); the complexity of the causes of RIF dictates the need to develop individualized treatment plans in clinical practice.

Successful implantation requires synchronization of the embryo and maternal endometrial development. Despite using good-quality euploid blastocysts, implantation failure occurs in approximately 32%-51% of embryos at the time of transfer (7). One potential cause of implantation failure is endometrial receptivity abnormalities (2, 8), mainly manifested by window of implantation (WOI) displacement and/or pathological disruption of the endometrium (9, 10). The main methods commonly used to assess endometrial receptivity are serum estrogen and progesterone levels and ultrasound morphological assessment (9). However, these methods currently have limitations such as low specificity and limited predictive value. Therefore, finding an effective diagnostic tool to objectively and accurately identify WOI for personalized embryo transfer (PET) is essential to improve pregnancy outcomes in patients with RIF. In recent years, with the development of genomics and high-throughput sequencing technologies, ERA has become a novel diagnostic method to objectively assess endometrial receptivity from a molecular perspective (11). ERA requires an endometrial biopsy taken at a specific period of menstruation to analyze gene expression and classify the results of the sample as pre-receptive, receptive, or post-receptive (12). According to the endometrial receptivity status measured by ERA, the patient adjusts the duration of progesterone exposure in the next cycle, and PET is performed to optimize embryo and endometrial synchronization. The effectiveness of ERA in enhancing clinical outcomes has not been convincingly established. While certain earlier investigations have indicated that PET can enhance clinical outcomes for patients experiencing RIF (13, 14), there are also studies that have determined that PET does not significantly improve pregnancy outcomes for this patient group (15, 16). On the other hand, Ruiz-Alonso et al. showed that the rate of non-receptive endometrium is higher in women with RIF than in those without RIF (17).

In order to observe the clinical application value of ERA in RIF population, this study investigated the effect of ERA-adjusted PET on pregnancy outcomes of FET cycles in RIF patients, and further analyzed the efficacy of ERA in patients with different number of previous implantation failures. Our study also compared the clinical specificities of receptive and non-receptive patients and analyzed whether there were underlying etiologies affecting WOI displacement.

## Materials and methods

### Study design

**Figure 1:**
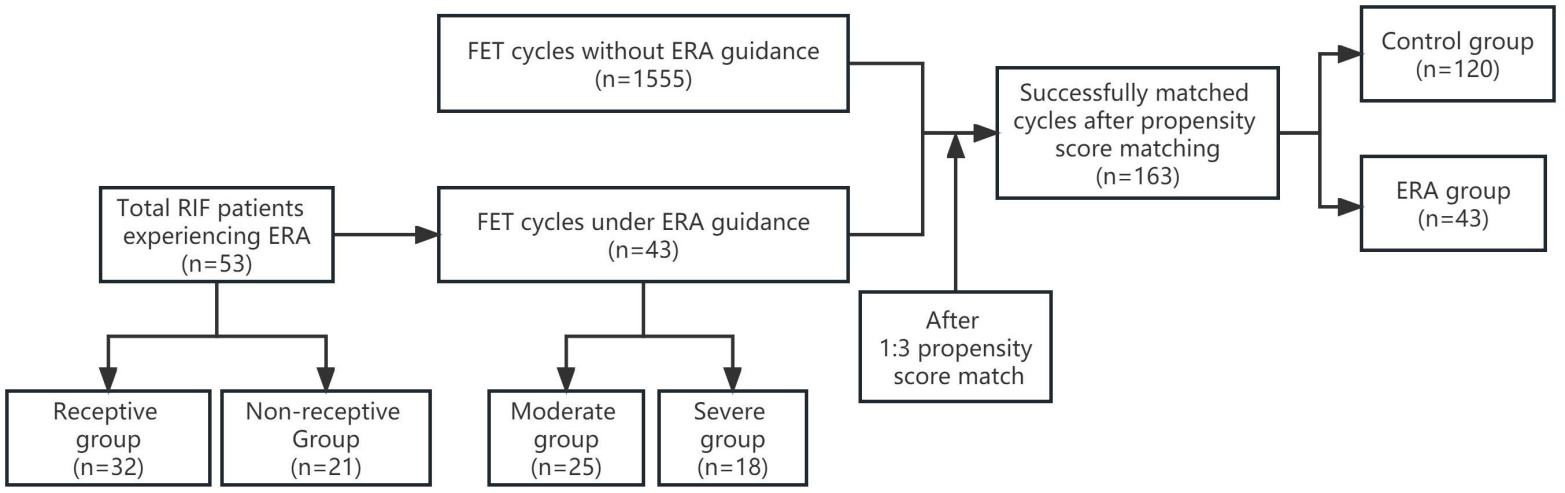
The flow chart of the study. ERA, endometrial receptivity analysis; FET, frozen embryo transfer; RIF, recurrent implantation failure.

Retrospective analysis of clinical data of patients with RIF who underwent FET at the Third Affiliated Hospital of Zhengzhou University Reproductive Medicine Center from January 2019 to December 2021.RIF is defined as the absence of implantation after two consecutive cycles. The cumulative number of transferred embryos was no less than four for cleavage-stage embryos and no less than two for blastocysts (4). Cycles in which PET was performed according to ERA results served as ERA group, and cycles in which conventional FET was performed as control group. The same patient treated with multiple cycles of freeze-thaw between January 2019 and December 2021 was included in only their first cycle. Patients in the ERA and control groups were matched 1:3 using propensity score matching (PSM). The main observations were the clinical pregnancy rate. The patients in the ERA group were further divided into a moderate group (previous number of cycles without achieving implantation <4) and a severe group (previous number of cycles without achieving implantation ≥4) according to the number of previous failures, and the effect of PET on pregnancy outcomes in both groups was analyzed. In addition, the ERA test results were divided into pre-receptive, receptive, and post-receptive periods, and we included the receptive in the receptive group and the pre-receptive and post-receptive periods in the non-receptive group according to the ERA test results. The baseline characteristics of the two groups and the differences in the underlying disease between the two groups of patients were analyzed to search for factors that may contribute to WOI displacement (**Figure 1**).

### Participants

Inclusion criteria: (i) Experienced at least 2 fresh or FET cycles of embryo transfer and cumulative transfer of at least 4 high-quality cleavage-stage embryos or 2 blastocysts without obtaining a clinical pregnancy; (ii) Age < 40 years; (iii) FET cycle. Exclusion criteria: (i) Oocyte donation for pregnancy; (ii)Severe chromosomal abnormalities in either the male or female partner; (iii) Severe endocrine, immune, and coagulation abnormalities that have not been corrected to normal, etc.; (iv) Patients with the incomplete data recording.

### ERA and PET

The formulation of FET protocol for patients is mainly based on the regularity of menstrual cycle and previous ovulation. For those patients with regular menstrual cycle and no ovulation disorder, transvaginal ultrasound was used to monitor follicular development and endometrial conditions from the 9th to 12th day of the menstrual cycle. When the dominant follicle was ≥18 mm, estradiol (E_2_) ≥550 pmol/L, and luteinizing hormone (LH)<10 U/L, human chorionic gonadotropin (β-HCG) was given to induce ovulation. Vaginal ultrasound monitoring was continued, and progesterone was administered starting on the day of ovulation to transform the endometrium: Oral dydrogesterone tablets (Dydrogesterone, Sauvage Pharmaceutical Co., the Netherlands, 10 mg/tablet) 10 mg twice daily, and 8% progesterone vaginal sustained-release gel (Serovar, Merck Serono Co., Germany, 90 mg/tablet) 90 mg once daily, 90 mg vaginal or progesterone soft capsules (Angeltam, France, France). 100 mg/pill), 200 mg twice daily, vaginally. A small sample of endometrial tissue was aspirated from the base of the uterus using an endometrial sampler on day 5 after transformation for detection. For those with irregular menses or ovulatory disorders, estradiol valerate (Progyla, Bayer Healthcare GMBH, Germany) is given orally 2-3 mg two to three times a day, combined with endometrial thickness during previous ovulatory periods. Estrogen was used for at least 10 days. When endometrial thickness >7 mm and E_2_ ≥100 ng/L, endometrial transformation began, and the pattern of progesterone transformation was consistent with the natural cycle. Similarly, a small sample of endometrial tissue was aspirated from the bottom of the uterus using an endometrial sampler on day 5 after transformation for detection. The steps of the endometrial biopsy were: washing the surface of endometrial tissue with sterile saline and then quickly put into liquid nitrogen, and then transferring it to a -80°C refrigerator for subsequent ribonucleic acid ( RNA) extraction. Extract RNA, reverse transcribe RNA to synthesize complementary deoxyribonucleic acid (cDNA), construct cDNA library, library quality control, and perform high-throughput sequencing. The data were analyzed using Chromgo, a computerized prediction program that gives "receptive" or "non-receptive" results for the endometrium examined, and "non-receptive" is further divided into "pre-receptive" and "post-receptive.” For patients with an ERA result of "receptive" endometrium, FET is performed at that time point in the next identical cycle, while for patients with "non-receiving" endometrium, the timing of embryo transfer will be adjusted according to the ERA results.

### Observation indicators

Clinical pregnancy was defined as the observation of one or more gestational sac on transvaginal ultrasonography 4 to 5 weeks after embryo transfer; Those with spontaneous termination of pregnancy at gestational age <24 weeks were considered spontaneous abortions; Live birth was defined as a newborn with one of four vital signs of heartbeat, breathing, umbilical cord pulsation, and muscle tension after delivery at 28 weeks of gestation or birth weight of 1000 grams or more. The pregnancy outcomes, including embryo implantation rate, clinical pregnancy rate, spontaneous abortion rate, and live birth rate, were compared between the two groups. Embryo implantation rate = total number of gestational sac transferred/total number of embryos transferred ×100%; Clinical pregnancy rate = number of clinical pregnancy cycles/total number of transplantation cycles ×100%; Spontaneous abortion rate = number of spontaneous abortion cycles/number of clinical pregnancy cycles × 100%; Live birth rate = number of cycles of live birth delivered at ≥28 weeks of gestation after transplantation/total number of transplant cycles ×100%.

### Statistical methods

To adjust for confounding factors associated with pregnancy outcomes, PSM was performed; the variables in the PSM include age, body mass index (BMI), years of infertility, type of infertility, basal follicle-stimulating hormone (FSH), endometrial thickness on the day of transfer, number of previous transfer failures, endometrial preparation protocol, number of embryos transferred, type of embryos transferred, and number of quality embryos transferred. To optimize the precision of the study, PET patients were matched to non-PET patients in a 1:3 matching ratio. The majority of PET patients were successfully matched with 3 non-PET patients. Finally, 43 PET patients were matched to 120 non-PET patients.

Normality assumptions for continuous variables were tested using the Kolmogorov-Smirnov test. For continuous variables that are approximately normally distributed, the mean ± standard deviation is used for statistical description, and for continuous variables that are not normally distributed, the median and interquartile spacing is used for statistical description. Student's t-test or Mann-Whitney test was used to compare statistical data between groups according to whether the data obeyed normal distribution. For categorical variables, the statistical analyses were performed using the chi-square test or Fisher's exact test for comparisons of outcomes. All tests were two-tailed; *P* < 0.05 was considered significant.

Sample size calculations were performed before the study was conducted to determine adequate statistical power for the study. A two-sided significance level of 0.05 and a power of 80%, calculated with PASS15, resulted in a minimum of 32 participants in the experimental group. The final control group of this study included a total of 43 study subjects, and the sample size of the study had sufficient statistical power.

## Results

### Basic patient information

From January 2019 to December 2021, a total of 53 RIF patients were treated with ERA in our center, of which 43 patients received FET cycle transplantation during this period, and the first PET cycle of these 43 patients was included in the ERA group, and the control group was finally included in 1555 cycles. A total of 120 cycles were matched as controls after 1:3 PSM. Before PSM, there was a statistical difference between the ERA and control groups in terms of years of infertility (4.40 ± 2.45 *vs*. 3.55 ± 2.77, *P* =0.042), type of infertility (55.8% *vs*. 37.4%,*P* =0.014), and number of previous graft failures (3.49 ± 1.37*vs*.2.14 ± 0.44, *P <*0.001). After PSM, there was no statistically significant difference in baseline information between the two groups (*P* > 0.05) (**Table 1**).

**Table 1:** Comparison of baseline information between the two groups before and after PSM.

Variables	Before PSM				After PSM				
	ERA Group	Control group	*t/χ*²	*P* value	ERA Group	Control group	*t/χ*²	*P* value	
Number of cycles	43	1555			43	120		
Age (years)	31.98 ± 3.11	32.29 ± 4.16	0.635	0.529	31.98 ± 3.11	32.06 ± 4.09	0.135	0.893
BMI (kg/m²)	23.13 ± 2.68	23.73 ± 3.03	1.297	0.195	23.13 ± 2.68	22.99 ± 2.98	-0.272	0.786
Number of years of Infertility (years)	4.40 ± 2.45	3.55 ± 2.71	-2.037	0.042	4.40 ± 2.45	4.28 ± 2.93	-0.248	0.804
Type of infertility (%)									
Primary infertility	55.8 (24)	37.4 (582)			55.8 (24)	56.7 (68)		
Secondary Infertility	44.2 (19)	62.6 (973)	6.009	0.014	44.2 (19)	43.3 (52)	0.009	0.923
Basic FSH (IU/L)	6.56 ± 2.15	6.65 ± 2.76	0.204	0.838	6.56 ± 2.15	6.25 ± 2.90	-0.656	0.513
Endometrial thickness at Transplantation date (mm)	9.11 ± 1.13	9.31 ± 1.58	1.119	0.269	9.11 ± 1.13	9.09 ± 1.28	-0.062	0.951
Number of previous Transplant failures	3.49 ± 1.37	2.14 ± 0.44	-12.421	<0.001	3.49 ± 1.37	3.23 ± 1.12	-1.246	0.215
Endothelial preparation program (%)									
Natural cycle	30.2 (13)	32.23 (503)			30.2 (13)	38.5 (43)		
Artificial Cycle	44.2 (19)	41.9 (652)			44.2 (19)	40.0 (48)		
Artificial Cycle after down-regulation	9.3 (4)	7.6 (118)			9.3 (4)	3.3 (4)		
Ovulation-promoting cycle	16.3 (7)	18.1 (282)	0.349	0.95	16.3 (7)	20.8 (25)	3.057	0.383
Number of embryos Transferred (%)									
1	37.2 (16)	43.4 (675)			37.2 (16)	34.2 (41)		
2	62.8 (27)	56.6 (880)	0.655	0.418	62.8 (27)	65.8 (79)	0.129	0.720
Type of embryos transferred (%)									
D3	18.6 (8)	34.3 (533)			18.6 (8)	25.8 (31)		
D5/D6	72.1 (31)	56.1 (873)			72.1 (31)	52.5 (63)		
D3+D5/D6	9.3 (4)	9.6 (149)	4.921	0.085	9.3 (4)	21.7 (26)	5.428	0.066
Number of quality Embryos transferred (%)									
0	37.2 (16)	37.5 (583)			37.2 (16)	45.8 (55)		
1	37.2%16)	39.4 (612)			37.2 (16)	33.3 (40)		
2	25.6 (11)	23.2 (360)	0.156	0.925	25.6 (11)	20.8 (25)	1.002	0.606

Data are expressed as mean ± standard deviation or frequency (%). PSM, propensity score matching; ERA, endometrial receptivity analysis; BMI, body mass index; FSH, follicle-stimulating hormone; D3, Embryos on day 3; D5, Blastocyst on day 5; D6, Blastocyst on day 6.

### Comparison of pregnancy outcomes between the two groups

**Figure 2:**
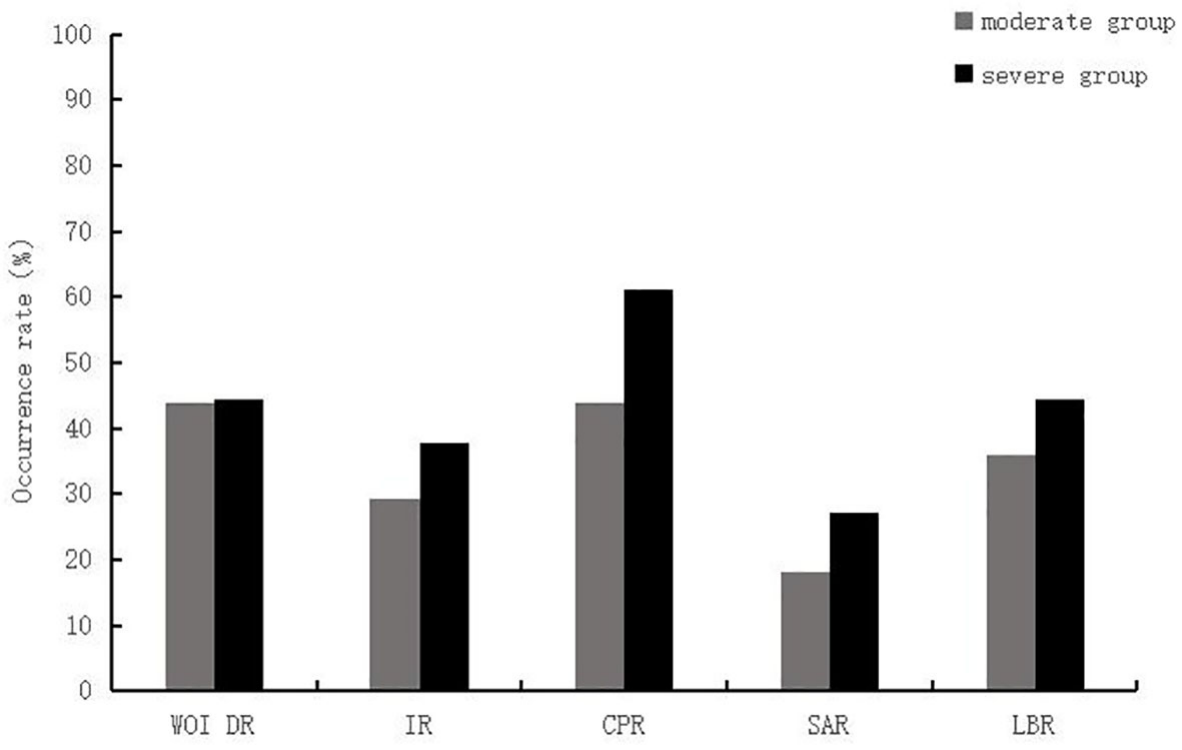
Comparison between the moderate and severe RIF group of patients in the ERA group. WOI DR, window of implantation displacement rate; IR, implantation rate; CPR, Clinical Pregnancy Rate; SAR, Spontaneous abortion rate; LBR, Live birth rate.

In terms of clinical outcomes, the differences between the ERA group and control patients after PSM were not statistically significant (*P* > 0.05) in terms of embryo implantation rate (32.9% *vs*. 34.7%), clinical pregnancy rate (51.2% *vs*. 48.3%), spontaneous abortion rate (22.7% *vs*. 20.7%), and live birth rate (39.5% *vs*. 38.3%) than in the control group (**Table 2**). Patients in the ERA group were further divided into severe (n=18) and moderate (n=25) groups according to the number of previous embryo transfer failures. There were no significant differences in WOI shift rate (44.0% *vs* 44.4%), embryo implantation rate (29.3% *vs* 37.9%), clinical pregnancy rate (44.0% *vs* 61.1%), spontaneous abortion rate (18.2% *vs* 27.3%) and live birth rate (36.0% *vs* 44.4%) between the two groups (**Figure 2**).

**Table 2:** Comparison of clinical outcomes between the two groups of patients after PSM.

Variables	ERA Group	Control group	*t/χ*²	*P* value
Implantation Rate (%)	32.9 (23)	34.7 (69)	0.076	0.783
Clinical Pregnancy Rate (%)	51.2 (22)	48.3 (58)	0.101	0.750
Spontaneous Abortion Rate (%)	22.7 (5)	20.7 (12)	0.040	0.842
Live Birth Rate (%)	39.5 (17)	38.3 (46)	0.019	0.890

Data are expressed as mean ± standard deviation or median and interquartile spacing, frequency (%). ERA, endometrial receptivity analysis.

### Comparison of clinical characteristics of patients in the receptive and non-receptive phases

Of the 53 patients who underwent ERA testing, 39.62% had ERA results of WOI displacement, of which 80.95% showed a pre-receptive status and 19.05% showed a post-receptive status. Comparing the clinical characteristics of the two groups, the age, BMI, years of infertility, type of infertility, basal FSH, basal LH, basal E_2_, basal progesterone, endometrial thickness on the transplantation date, number of previous transplantation failures, whether infertility was due to male factor, whether infertility was due to pelvic tube factor, whether combined with endometriosis, whether combined with endometrial polyps, whether combined with polycystic ovary syndrome in both groups The differences were not statistically significant (*P* > 0.05).

## Discussion

In this retrospective cohort study, we used PSM to reduce confounding bias. The results of our study showed that PET performed under ERA guidance did not significantly improve pregnancy outcomes in RIF patients with FET cycles. To clarify whether the number of previous implant failures of patients affects pregnancy outcomes after PET, we performed a subgroup analysis of ERA patients according to the number of previous implant failures and divided them into a severe group and a moderate group, which showed no statistical difference in WOI excursion rate and pregnancy outcome between the two groups. In this study, we further analyzed the factors affecting WOI displacement, divided the patients into receptive and non-receptive groups based on the ERA test results, and analyzed the clinical characteristics of the two groups, which showed no statistically significant differences between the clinical characteristics of the two groups.

In this study, 39.62% of the RIF patients who underwent ERA testing experienced a WOI displacement, and of those who experienced a displacement, 80.95% exhibited a pre-receptive state, and 19.05% exhibited a post-receptive state. There was no significant difference between the receptive and non-receptive groups in terms of the number of previous implant failures, infertility factors, the prevalence of endometriosis (EMS), polycystic ovarian syndrome (PCOS), and endometrial polyps. The results of many studies have shown that diseases such as PCOS and EMS affect endometrial receptivity (ER) (18–22). The significant decrease in ER in PCOS patients is closely related to the regulatory mechanisms of oxidative stress, metabolic abnormalities, endocrine disruption, and other mechanisms in the endometrium (23, 24). Patients with EMS with infertility have a variety of abnormally expressed factors related to endometrial receptivity in the endometrium *in situ*, and EMS patients often have varying degrees of decreased endometrial receptivity (25). Previously, Mahajan et al. tested ERA in patients with a history of previous implant failure, and their findings showed that EMS patients were more likely to have a displaced WOI (26). But our study results showed a similar prevalence of EMS in both groups. We have a different population scope than Mahajan et al. study. Further exploration is needed regarding whether EMS causes WOI displacement.

The results of our study showed no significant difference in pregnancy outcomes of patients between the ERA and control group. In 2013 a small prospective study conducted found that PET improved pregnancy outcomes in patients with RIF (17). The results of the 5-year multicenter randomized controlled trial study published by Carlos Simón et al. in 2020 showed a significantly higher cumulative live birth rate in patients who received PET compared to controls after 12 months of follow-up in a study population of patients undergoing their first embryo transfer (14). A recent systematic review published by Arian et al. showed no significant difference in pregnancy outcomes between patients in the PET and non-PET groups, and subgroup analysis of three studies with populations of patients with RIF showed that ERA did not significantly improve pregnancy outcomes (27). We divided the patients into a moderate RIF group and a severe RIF group according to the number of previous implant failures to explore whether PET is more valuable for patients with more previous implant failures. The results showed that there was no statistically significant difference in the rate of WOI displacement in the moderate group compared with the severe group, and there was no significant difference in pregnancy outcome between the two groups. Previous studies have found that WOI displacement occurs in a proportion of patients with a good prognosis, but in RIF, it occurs at a higher rate (17). Our study was analyzed for the RIF population, and the results did not show a greater value of PET for RIF patients with a greater number of previous implant failures.

The complexity and diversity of the etiology of RIF require the clinician to develop an individualized treatment plan for each etiology of the patient, and WOI displacement may only be part of the etiology of embryo implantation failure in RIF patients, as maternal status and embryo ploidy can affect embryo implantation (1, 2, 4, 7). In 2021 a retrospective study showed that the combination of ERA and endometrial immunoblots is more likely to be of clinical value than ERA or immunoblots alone (28). Our study excluded populations with immune abnormalities, but our study did not ensure that all transferred embryos were in a haploid state. Violeta Fodina et al. showed that RIF patients could benefit from the use of the preimplantation genetic testing (PGT-A) method to detect embryonic aneuploidy, but the ability of ERA testing to improve clinical outcomes in intracytoplasmic sperm injection (ICSI) cycles appears to be rather limited (29). A study by Mauro Cozzolino et al. showed that PGT-A may be beneficial in patients with moderate recurrent implantation failure but not in severe cases and that ERA has no clinical benefit in patients with RIF (30). In clinical practice for patients with RIF, ERA is currently available as a complementary test after excluding embryonic factors and other maternal etiologies.

The advantage of this study is that the use of PSM makes the study more comparable. Real-world studies (RWS) are susceptible to confounding factors that make the credibility of their findings questionable, while PSM can better address the issue of comparability between the RWS treatment and control groups (31). We explored whether PET is more valuable in patients with a higher number of previous implant failures and, in addition, analyzed the effect of the patient’s underlying disease on WOI displacement to guide the clinical application of ERA.

A limitation of this study is that partial endometrial damage can occur during ERA endometrial sampling, and a recent meta-analysis showed that the effect of endometrial damage on the live birth rate is not known (32). Therefore, it is unclear whether endometrial damage as part of the ERA procedure would have any impact on the pregnancy outcomes we observed. On the other hand, technology is rapidly advancing, and some scholars have suggested that the accuracy of next-generation sequencing (NGS) technology may surpass that of array sequencing. Previous research findings should be re-validated using NGS to avoid discrepancies in results across different studies. With the continuous updates in ERA technology, further research is needed to determine whether it is possible to more sensitively and accurately determine the Window of Implantation (WOI), thereby significantly improving pregnancy outcomes. In the future, more accurate detection methods may emerge to enhance the clinical application value of ERA. Additionally, proteomics and microbiomics may also become reliable tools for studying the WOI. Our study is a non-large sample size retrospective study, ERA is not a routine test in assisted reproductive technology clinics, and previous articles on the effectiveness of ERA and potential factors influencing WOI migration are limited and not conclusive at this time. Future prospective randomized controlled trials with large sample sizes are needed to assess the value of ERA in clinical practice.

## Conclusion

In our study, PET did not significantly improve pregnancy outcomes in patients with RIF, no significant differences in pregnancy outcomes were found between patients with moderate and severe RIF after PET, and no underlying etiology affecting WOI displacement was identified. Patients undergoing ERA testing need to undergo invasive testing to obtain the tissue samples for the ERA test, and patients are not available for embryo transfer in the month of testing, which results in longer patient treatment times, increased medication use during treatment, and additional invasive medical interventions. On the other hand, ERA is an additional financial burden for the patient. ERA is still in the research stage, but its accuracy in predicting WOI and the improvement effect on clinical outcomes are not clear, so it is not recommended as a routine treatment for RIF patients.

## Data Availability

The raw data supporting the conclusions of this article will be made available by the authors, without undue reservation.
